# Moderate Hypofraktionierung beim Harnblasenkarzinom – ein neuer Versorgungsstandard?

**DOI:** 10.1007/s00066-021-01804-2

**Published:** 2021-07-06

**Authors:** Oliver J. Ott

**Affiliations:** Strahlenklinik, Universitätsklinikum Erlangen, Universitätsstr. 27, 91054 Erlangen, Deutschland

## Hintergrund und Ziel

Im englischsprachigen Raum werden lokal fortgeschrittene Harnblasenkarzinome üblicherweise alternativ mit 64 Gy in 32 Fraktionen über 6,5 Wochen oder mit 55 Gy in 20 Fraktionen über 4 Wochen organerhaltend bestrahlt. Auf der Basis vorliegender Daten wurde eine vergleichbare Effektivität und Verträglichkeit beider Schemata angenommen, ein direkter Vergleich beider Fraktionierungen wurde jedoch bislang noch nicht vorgenommen bzw. publiziert.

## Patienten und Methoden

Zu diesem Zweck wurden die Daten von zwei randomisierten britischen Multicenterstudien (BC2001: Radiotherapie ±5-FU und Mitomycin C, NCT00024349; BCON: Radiotherapie ± hypoxiemodifizierende Therapie, NCT00033436) gepoolt und analysiert [1]. In die Metaanalyse wurden pT1G3- sowie T2-4N0M0-Harnblasenkarzinome eingebracht. Die Fraktionierung wurde in beiden Studien gemäß den geltenden lokalen Behandlungsstandards gewählt. Primäre Endpunkte der Metaanalyse waren die invasive lokoregionale Kontrolle sowie die Spättoxizität an Harnblase und Rektum.

## Ergebnisse

Aus den beiden Studien konnten 782 Patient*innen einer Fraktionierung zugeordnet werden; 376/782 (48 %) erhielten 64 Gy in 32 Fraktionen und 406/782 (52 %) 55 Gy in 20 Fraktionen. Die mediane Nachbeobachtungszeit betrug 120 Monate (IQR 99–159). Patient*innen mit 55 Gy in 20 Fraktionen hatten ein vergleichsweise niedrigeres Risiko für ein invasives lokoregionales Rezidiv (adjustierte Hazard-Ratio 0,71 [95 % CI 0,52–0,96]). Bezüglich der Toxizität fanden sich keine Unterschiede (adjustierte Risikodifferenz −3,37 % [95 % CI −11,85 bis 5,10]).

## Schlussfolgerung der Autoren

Die moderate Hypofraktionierung mit 55 Gy in 20 Fraktionen war der konventionellen Fraktionierung hinsichtlich invasiver lokoregionaler Tumorkontrolle sowie Toxizität nicht unterlegen („non-inferiority“), bezüglich invasiver lokoregionaler Kontrolle sogar überlegen. Daher sollte die moderate Hypofraktionierung mit 55 Gy in 20 Fraktionen für Patienten mit einem lokal fortgeschrittenen Harnblasenkarzinom als neuer Versorgungsstandard übernommen werden.

## Kommentar

Die Autoren präsentieren hier eine sehr interessante Metaanalyse zur Fraktionierung der Radiotherapie (RT) beim lokal fortgeschrittenen Transitionalzellkarzinom der Harnblase mit dem Anspruch, einen neuen Therapiestandard zum organerhaltenden Vorgehen zu definieren. Die Daten wurden in zwei randomisierten Studien [2, 3] prospektiv gesammelt und nach allen Regeln der Kunst zusammen analysiert. Auch die Fallzahl imponiert. Es stellen sich allerdings einige Bedenken ein, ob die beschriebene Überlegenheit des moderat hypofraktionierten Vorgehens auch außerhalb der britischen Inseln als neuer Standard übernommen werden kann:

1. In der heutigen Zeit erwartet man als Anlass für eine weitreichende Änderung eines erprobten Therapiestandards einen direkten randomisierten Vergleich (oder noch besser mehrerer randomisierter Studien), wie z. B. bei der Etablierung der Hypofraktionierung bei der Radiotherapie des Mammakarzinoms der Frau. Im vorliegenden Fall handelt es sich zwar um Patient*innen aus randomisierten Studien, jedoch ging es in beiden Studien um einen Vergleich mit und ohne Radiosensibilisierung. Die beiden genannten Fraktionierungsschemata wurden in insgesamt 50 teilnehmenden Zentren je nach lokaler Präferenz gewählt und die vorliegende Metaanalyse ungeplant durchgeführt. Um die dabei entstehenden Unsicherheiten zu kompensieren, nahm man allerhand Adjustierungen vor. Es handelt sich letztlich also hinsichtlich der Fraktionierungen nicht um einen direkten und prospektiv geplanten Vergleich.
2. Im Vergleich mit der in anderen Ländern etablierten trimodalen Therapie [4–6] erscheint uns die erreichte invasive lokoregionale 5‑Jahres-Rezidivrate mit 28 % doch ziemlich hoch. Grund dafür scheint uns die Tatsache zu sein, dass die obligate kurativ intendierte transurethrale Resektion (TUR) mit dem Ziel, vor der RCT in den repräsentativen Rand- und Grundproben eine R0-Resektion zu erreichen, im Vereinigten Königreich nicht flächendeckend vorgesehen war.
3. Ein weiterer kritischer Punkt ist zu bedenken: Postoperativ gilt die alleinige Radiotherapie gegenüber der simultanen RCT als unterlegen und sollte laut der aktuellen Leitlinien auch nicht mehr vorgenommen werden, wenn der/die Patient*in fit für eine begleitende Chemotherapie ist [7, 8]. In Kontinentaleuropa und in den Vereinigten Staaten von Amerika wurde und wird dabei bevorzugt eine Cisplatin-basierte Radiochemotherapie empfohlen [4]. Da aber mit der Hypofraktionierung eine Verkürzung der Gesamtbehandlungszeit auf vier Wochen verbunden ist, kann die notwendige simultane Chemotherapie mit der in Deutschland verbreiteten Applikation in der ersten und fünften Bestrahlungswoche nicht mehr realisiert werden. Vor diesem Hintergrund erscheint es sehr unwahrscheinlich, dass der in unserer hier kommentierten Studie beschriebene Vorteil der lokalen Tumorkontrolle durch eine moderate Hypofraktionierung erreichbar wäre. Sicherlich gäbe es dafür Lösungen, aber bisher jedenfalls keine Daten. Und es ist letztlich auch unklar, ob eine Radiochemotherapie mit 5‑FU und Mitomycin C einer Cisplatin-basierten RCT gleichwertig ist, denn auch hier fehlt ein entsprechender randomisierter Vergleich.
4. Schließlich darf nicht übersehen werden, dass in den beiden britischen Studien nur eine mehr oder weniger entleerte Harnblase mit einem 1,5 cm breiten Sicherheitssaum bestrahlt wurde ohne Einschluss der pelvinen Lymphabflussgebiete, die zumindest in Deutschland elektiv traditionell mitbestrahlt werden [7]. Es ist deshalb auch hier fraglich, ob die Gleichwertigkeit der beiden Fraktionierungsschemata insbesondere hinsichtlich der Toxizität auch dann noch gegeben wäre.

## Fazit

Zweifelsfrei handelt es sich bei der diskutierten Metaanalyse um einen wertvollen Beitrag in der Diskussion zur Optimierung der organerhaltenden Therapie beim Harnblasenkarzinom. Wie bei anderen Entitäten, z. B. Mamma- und Prostatakarzinom, könnte die moderate Hypofraktionierung auch beim Harnblasenkarzinom für die Patient*innen Vorteile mit sich bringen. Jedoch lässt es die aktuelle Datenlage nicht zu, auch außerhalb des Herrschaftsgebietes der Windsors von einem neuen Standard zu sprechen.

*Oliver J. Ott, Erlangen*
